# Supplementary material for: Design of a bi-directional methodology for automated assessment of compliance to continuous application of clinical guidelines, and its evaluation in the type 2 diabetes domain
Source: PLoS One. 2024 May 20;19(5):e0303542. doi: 10.1371/journal.pone.0303542 (PMC11104637; doi:10.1371/journal.pone.0303542)
Supplement: S1 Appendix — (DOCX) [file pone.0303542.s001.docx]

# S1 Appendix

## The fuzzy temporal reasoner of the BIKBAC methodology

To better understand the motivation behind the use of fuzzy temporal logic in the compliance analysis process of the BIKBAC methodology, consider the following example of a *Clinical Quality Measure* (CQM) in the domain of the current study, i.e., Type II Diabetes: “Low Density Lipoprotein (LDL-C) Control in Diabetes Mellitus”, defined as “Percentage of patients aged 18 through 75 years with diabetes mellitus who had most recent LDL-C level in control (less than 100 mg/dL)”. A simplistic algorithm using this rigid cut-off value would assign a care provider who manages a group of 50 patients, whose LDL-C values at the point in which they were examined were all just slightly higher than 100 mg/dL, say 106, a quality measure of zero. Such an extreme assignment is not likely to be well received by the clinical community, and would decrease trust and acceptance of quality assessment results. However, due to the use of the fuzzy temporal logic mechanism to assess quality measures, the *DiscovErr* system would assess the quality of care of that group as quite high, say, by assigning them a membership score of, say, 0.88 or even 0.93, although the compliance would not be perfect. However, an LDL-C value of, say, 130 or higher would be assigned a membership score of 0, while intermediate values would be assigned some membership score between 0 and 1, using a linear or another membership function to interpolate between the two extreme values.

The reasoning process that is applied by the Fuzzy Temporal Reasoner involves a multistep process. In the following paragraphs, we describe each step of the reasoning process, and demonstrate it using an example for the evaluation of a Hypertension (High Blood Pressure) concept. For this example, we define Hypertension as a *State* concept, abstracted from two *Primitive* concepts, systolic blood pressure (SBP) and diastolic blood pressure (DBP), each is a numeric concept defined with a local persistency of one hour (i.e., the measurement is good for one hour). The abstraction includes the logic OR operator applied on two constraints on the values of the *Primitive* concepts. The definition of the Hypertension concept:

Hypertension = {SBP > 140 mmHg OR DBP > 90 mmHg}

Examples of the raw measurements of the patient’s systolic and diastolic blood pressures are illustrated in S1 Fig.

**S1 Fig.** **Blood Pressure measurements used for the demonstration of the Fuzzy Temporal Reasoner*.***

### Extrapolation of temporal intervals

The first step of the reasoning process includes extrapolation of temporal intervals using the *Temporal-Persistence* knowledge that was specified for the raw concepts. In addition, each pair of intervals of the same concept that share the same value, and share a time period (i.e., overlapping intervals), are merged into a single interval of this concept that is assigned the same value.

In the example (see S2 Fig), each blood pressure measurement is now extrapolated to create an interval of one hour, the three DBP measurements are extrapolated to three consequent intervals with the same value = 86. In the merging step, these three intervals are merged into a single longer interval with the same value.

**S2 Fig.** **Extrapolation of the time-stamped measurements by the Fuzzy Temporal Reasoner.**

### Temporal partitioning

The second step of the temporal reasoning process involves partitioning of the temporal data. The idea behind this operation is to create a segmented (partitioned) temporal representation, with the minimal set of partitions, in which each relevant concept has zero or a single value. This is done to support the next steps of the reasoning process, in which the evaluation logic is applied on each of these partitions.

In the example (see S3 Fig), the data is partitioned into five partitions. In the first partition, none of the concepts is provided with a value; in the second partition, the DBP value is 86, and the SBP value is 125; in the third partition, the DBP value is 86 and the SBP value is 139; in the fourth partition the DBP value is still 86 and the SBP has no value; and in the last partition, none of the concepts is provided with a value.

**S3 Fig.** **Temporal partitioning by the Fuzzy Temporal Reasoner*.***

### Fuzzy evaluation of the logical relations

The third step of the reasoning process involves the evaluation of the logical relations. The evaluation is done for each parameter value in each of the partitions.

In classic logic, the evaluation of logical relation results with a *true* or a *false* value, referred as the *truth-value* of a relation. In fuzzy logic, the result of a relation evaluation is called the membership score, and is represented as a continuous (rational) number, usually between 0 and 1. For that, we extended the representation of logical relations of the KBTA schema, with an attribute called a *deviation-interval*. This attribute is used in the relation evaluation to enable the assignment of a membership score in cases where the constraint is not fully satisfied. In such cases, a special *fuzzification-function* is applied for the reasoning process.

The *fuzzification-function* receives the following arguments: the *current*-*value* of the parameter, the *threshold*, the *deviation-interval,* and the *relation-operator* specified in the constraint definition*.* The *deviation-interval* represents the maximal deviation from the threshold that can be evaluated with a membership score higher than zero; thus, in cases where the absolute distance between the *current-value* and the *threshold* is greater than the *deviation-interval*, the membership score is evaluated as zero. In all other cases, the membership score is calculated according the following formula:

S4 Fig illustrates the application of the *fuzzification-function* for the evaluation of the constraint SBP > 140 mmHg, with a *deviation-interval* of 10 mmHg.

**S4 Fig. Illustration of the *fuzzification-function*.** Evaluation of the constraint SBP>140 mmHg, with a deviation-interval of 10 mmHg. On a measurement of SBP=139, the membership score is evaluated as 0.9; on a measurement of SBP=135, the membership score is evaluated as 0.5; on any measurement of SBP≤130, the membership score is evaluated as 0; on any measurement of SBP≥140, the membership score is evaluated as 1.

S5 Fig illustrates the complete logical constraint evaluation on each concept value in each partition that was generated in the previous step of the example. The interval with SBP=125 was evaluated with a membership score = 0, the interval with SBP=139 was evaluated with a membership score = 0.9, and each interval with DBP=86 was evaluated with a membership score = 0.6.

**S5 Fig. Evaluation of logical constraints by the Fuzzy Temporal Reasoner*.***

### Evaluation of logic operators

The last step of the reasoning process is the evaluation of the logic operators within compound logic expressions. For this, an additional fuzzy logic technique is used.

The operators ***AND****,* ***OR****,* and ***NOT*** of classic logic, exist in fuzzy logic with a different implementation. A fuzzy logic implementation of these logic operators, called *Zadeh operators,* uses the minimum function for the evaluation of the *AND* operator, and the maximum function for the evaluation of the *OR* operator, and uses the function 1-truth-value to evaluate the *NOT* operator.

#### AND and OR operators

In the Fuzzy Temporal Reasoner implementation we used the Zadeh operators for the evaluation of the ***AND*** and ***OR*** operators. The minimum function is used to evaluate the *AND* operator only in cases in which a membership score exists for all of the operands of the expression. The maximum function is used to evaluate the *OR* operator in cases in which the membership score is available for at least one of the operands of the expression.

S6 Fig illustrates evaluation of the logic operators on the demonstration example. The expression in the example is a compound expression using the *OR* operator (SBP>140 *OR* DBP>90); thus, for each of the partitions, the maximum function is applied on the membership scores that were calculated in the previous steps.

**S6 Fig. Evaluation of logic operators by the Fuzzy Temporal Reasoner.**

The evaluation of the logic operators as described above enables the Fuzzy Temporal Reasoner to evaluate any complex compound expression. The knowledge specified in the KBTA schema allows the representation of complex compound logic expressions, represented in the form of *AND-OR* trees. For example, a definition of the Preeclampsia diagnosis is “high blood pressure with proteinuria in a pregnant woman after 20 weeks of gestation”. Such definitions are specified in the knowledge as compound expressions, illustrated in S7 Fig using an *AND-OR* tree. The Fuzzy Temporal Reasoner uses a recursive implementation of the fuzzy logic evaluation function that supports evaluating any complex AND-OR tree.

**S7 Fig. AND-OR tree representation of the Preeclampsia diagnosis concept.**

#### The NOT operator

For the ***NOT*** operator we have implemented an operation that inverts the relation by replacing the relation’s operator with an opposite operator. For example, the expression *NOT*(*x ≥ y*) is evaluated as (*x < y*). The following example illustrates this new implementation. Consider the expression that defines a normal blood pressure condition as being *NOT*(*DBP* *>* 90 mmHg). The new implementation of the operator would set the (fuzzy) truth value to 1 whenever *DBP ≤ 90.* When the values are between 90 to 100, the truth value would be set between 1 and 0, and whenever DBP *≥* 100, the truth value would be set to 0. S8 Fig demonstrates this concept.

**S8 Fig.** **An example for fuzzy-evaluation of the NOT operator.** The left graph displays the evaluation of the constraint DBP > 90mmHg, with deviation interval of 10mmHg. The right side displays the evaluation of the false-value of the same constraint, i.e., NOT(DBP > 90mmHg), assuming the same deviation interval.

To evaluate the fuzzy **false-value of compound operations**, we used an implementation of De Morgan’s law. Evaluation of the expression *NOT(x OR y)*, is implemented as *(NOT(x) AND NOT(Y)).*

For example, to determine the fuzzy truth value of NOT having hypertension (which might be, for example, a stop condition for a therapy action), given the measurements of SBP=139 and DBP=92, with a deviation interval of 10mmHg, would be evaluated as:

*fuzzy-value(NOT((SBP ≥ 140) OR (DBP ≥ 90))) = fuzzy-value((SBP < 140) AND (DBP < 90)) = min(fuzzy-value(139), fuzzy-value(92)) = min(1,0.8) = 0.8*

Note that, assuming that the deviation interval for DBP ≥ 90mmHg is 10mmHg, the membership score of DBP=92 within the context of the fuzzy constraint DBP < 90, is 0.8 (see S8 Fig).
