# Supplementary figures and images for: Design of a bi-directional methodology for automated assessment of compliance to continuous application of clinical guidelines, and its evaluation in the type 2 diabetes domain
Source: PLoS One. 2024 May 20;19(5):e0303542. doi: 10.1371/journal.pone.0303542 (PMC11104637; doi:10.1371/journal.pone.0303542)

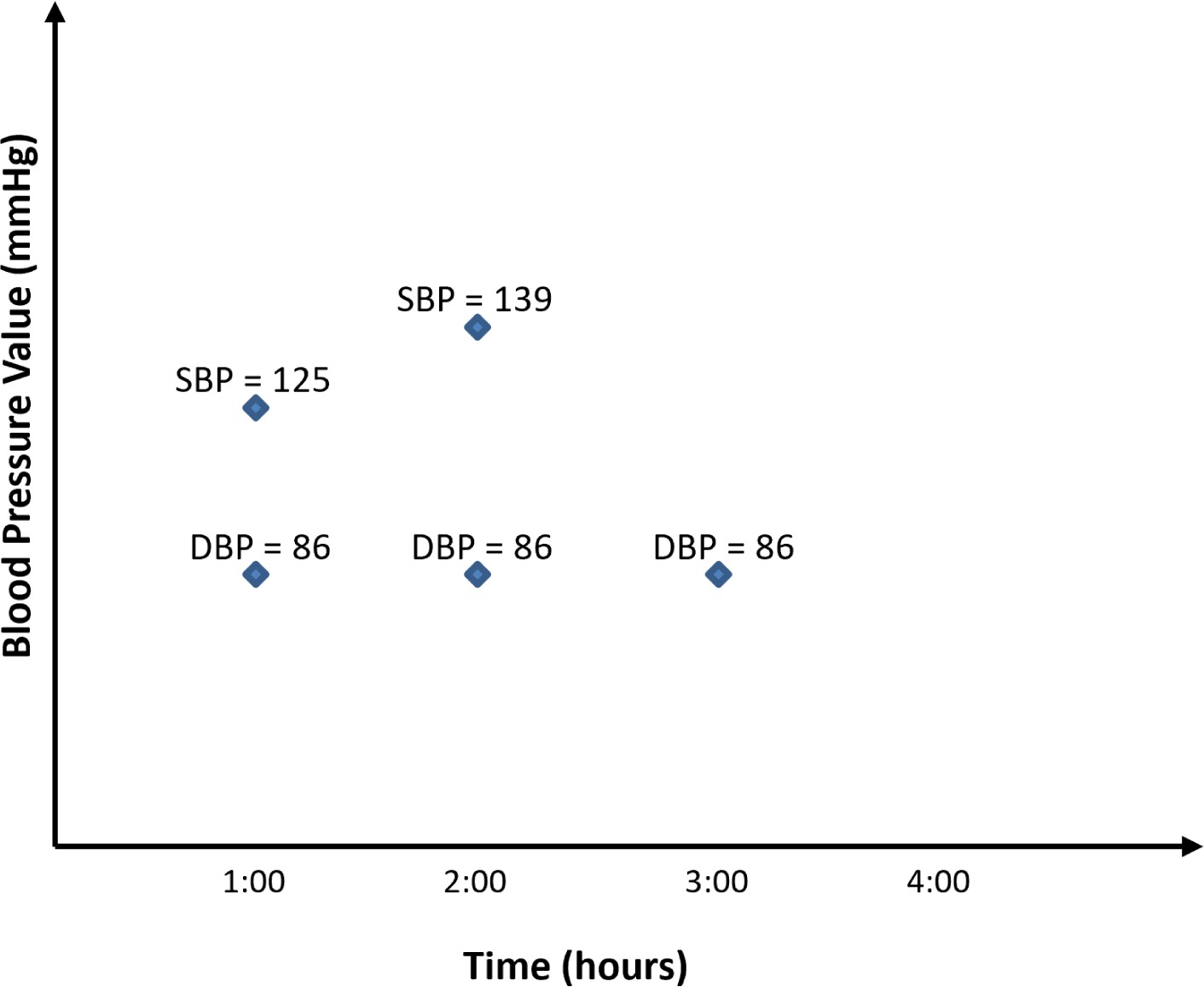

Supplement: S1 Fig — (TIF) [file pone.0303542.s002.tif]

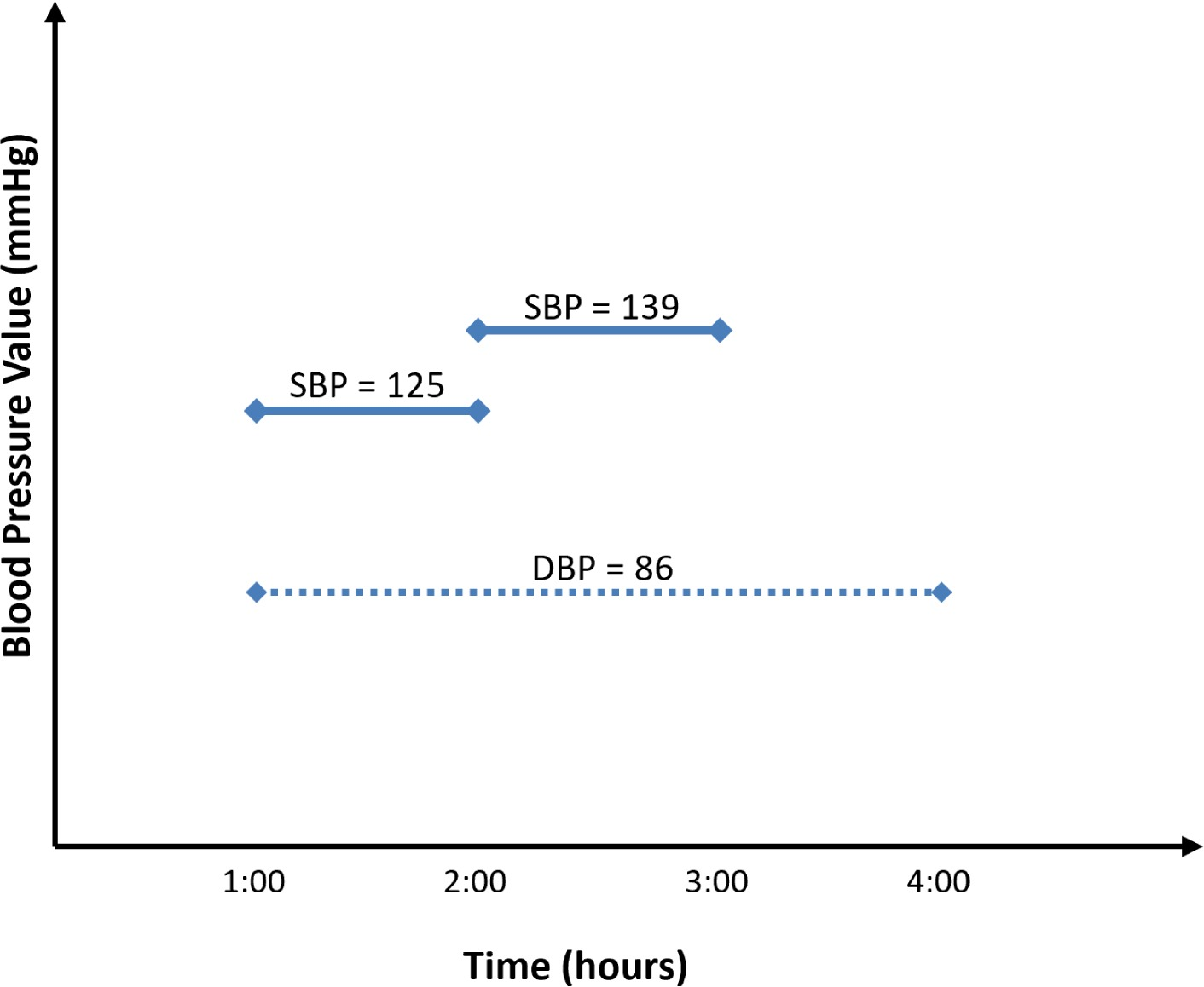

Supplement: S2 Fig — (TIF) [file pone.0303542.s003.tif]

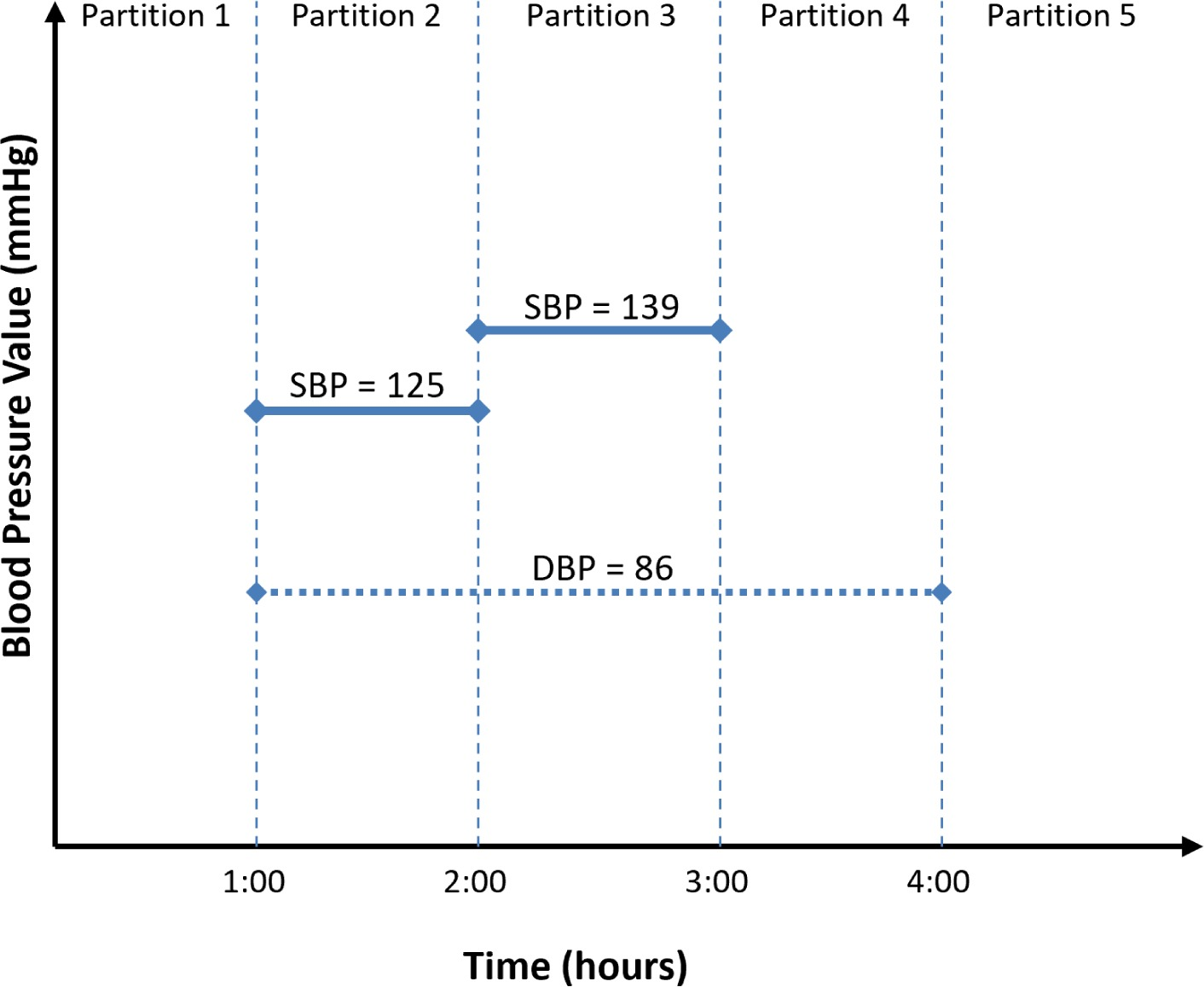

Supplement: S3 Fig — (TIF) [file pone.0303542.s004.tif]

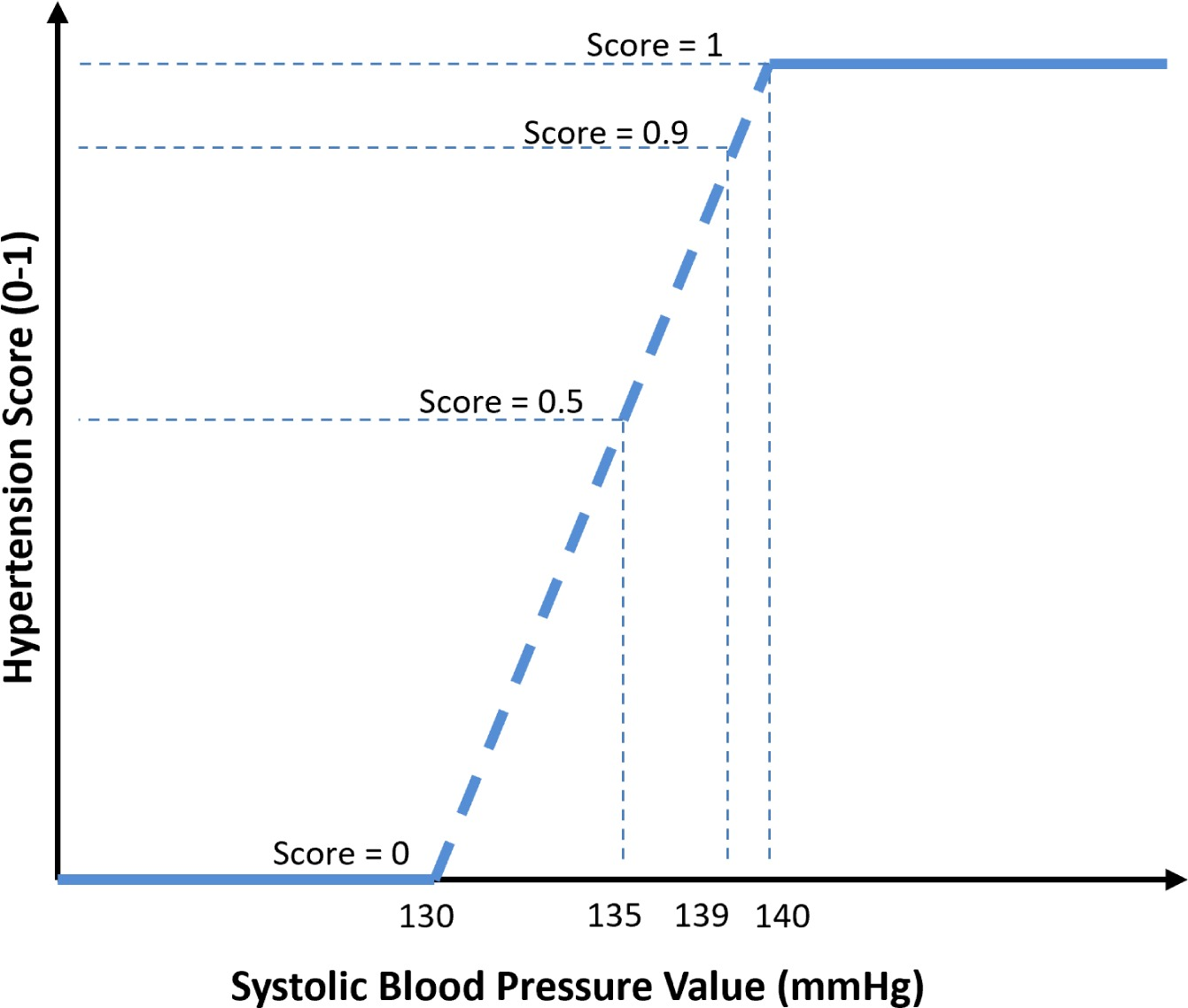

Supplement: S4 Fig — Evaluation of the constraint SBP>140 mmHg, with a deviation-interval of 10 mmHg. On a measurement of SBP = 139, the membership score is evaluated as 0.9; on a measurement of SBP = 135, the membership score is evaluated as 0.5; on any measurement of SBP≤130, the membership score is evaluated as 0; on any measurement of SBP≥140, the membership score is evaluated as 1. (TIF) [file pone.0303542.s005.tif]

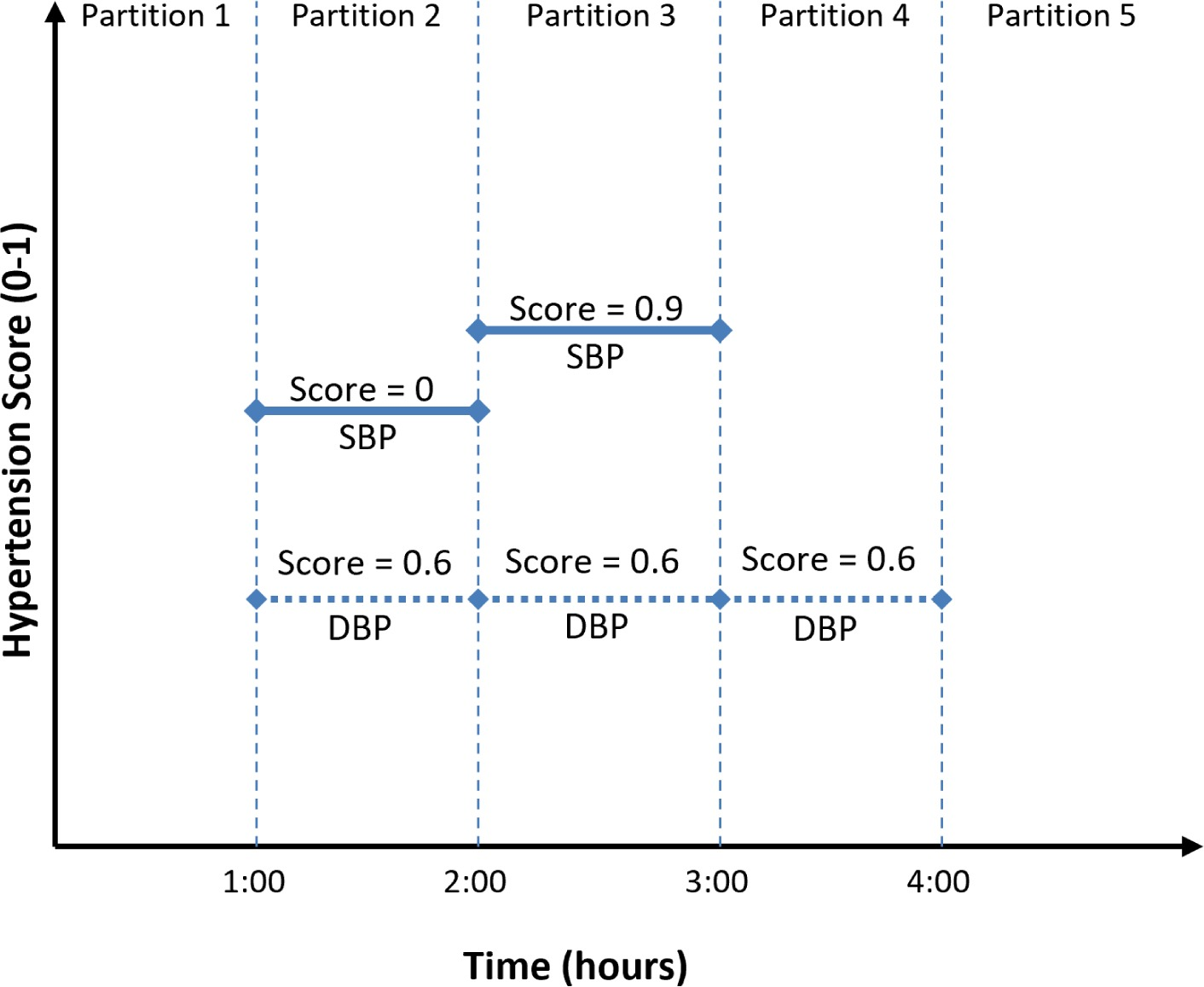

Supplement: S5 Fig — (TIF) [file pone.0303542.s006.tif]

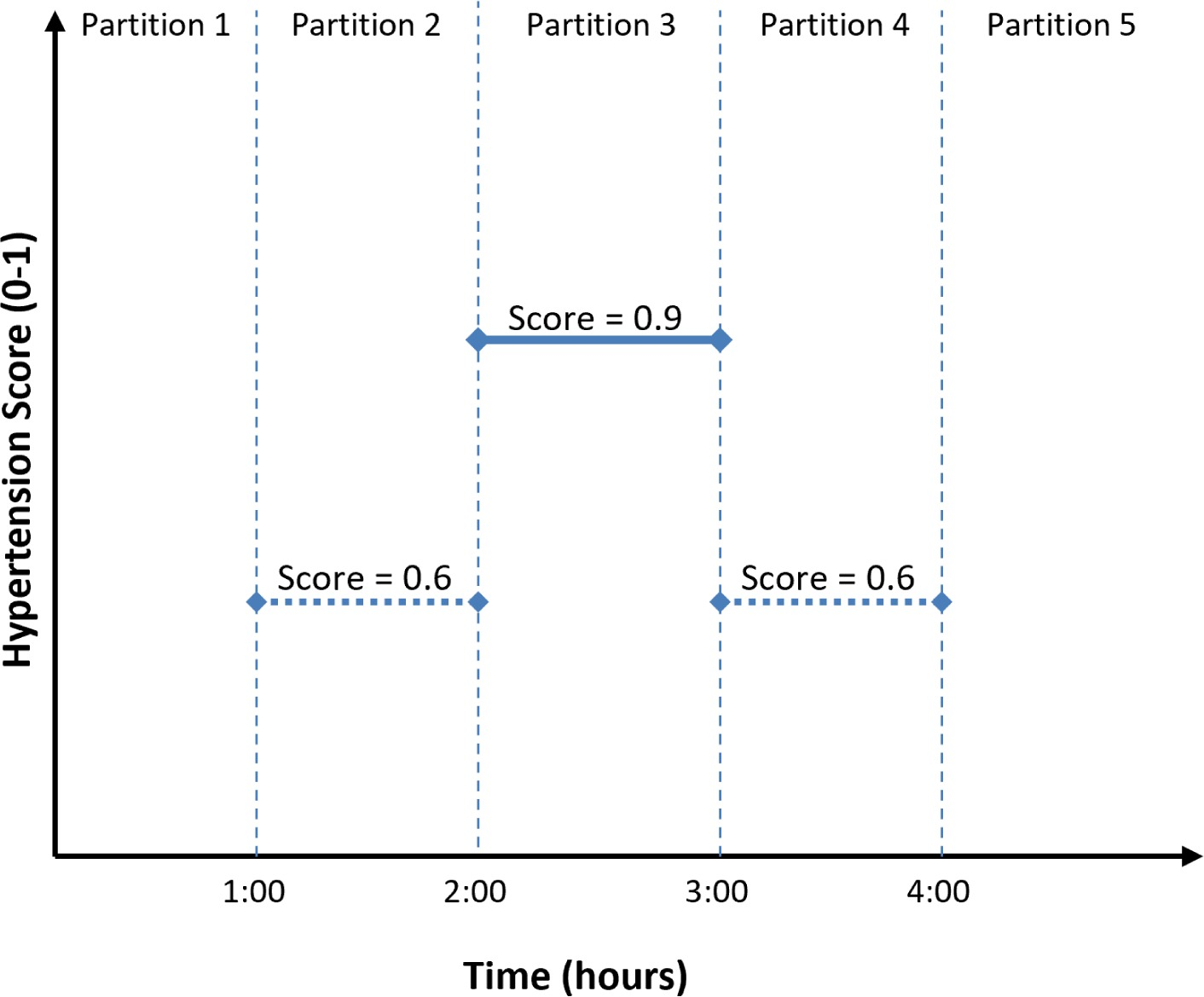

Supplement: S6 Fig — (TIF) [file pone.0303542.s007.tif]

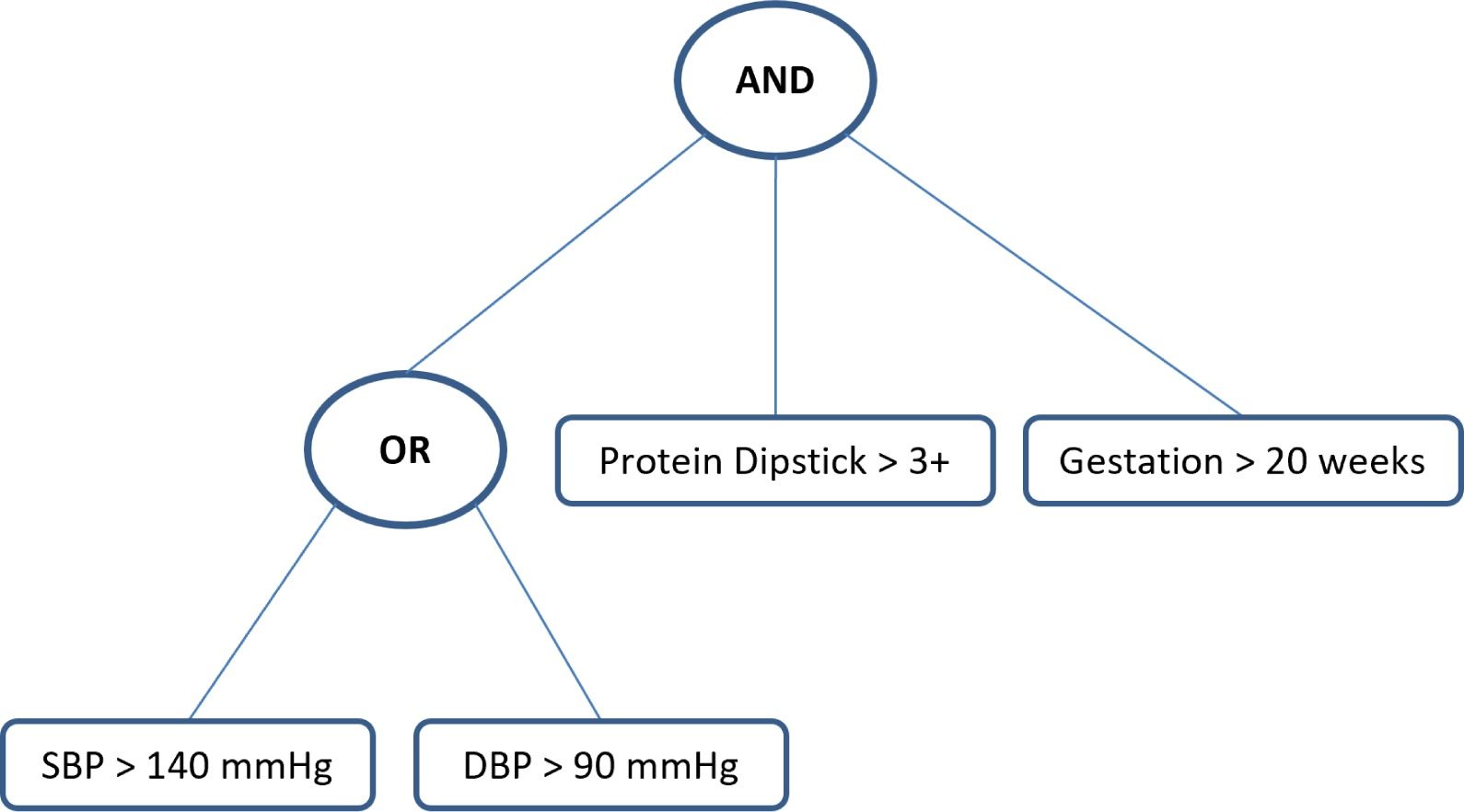

Supplement: S7 Fig — (TIF) [file pone.0303542.s008.tif]

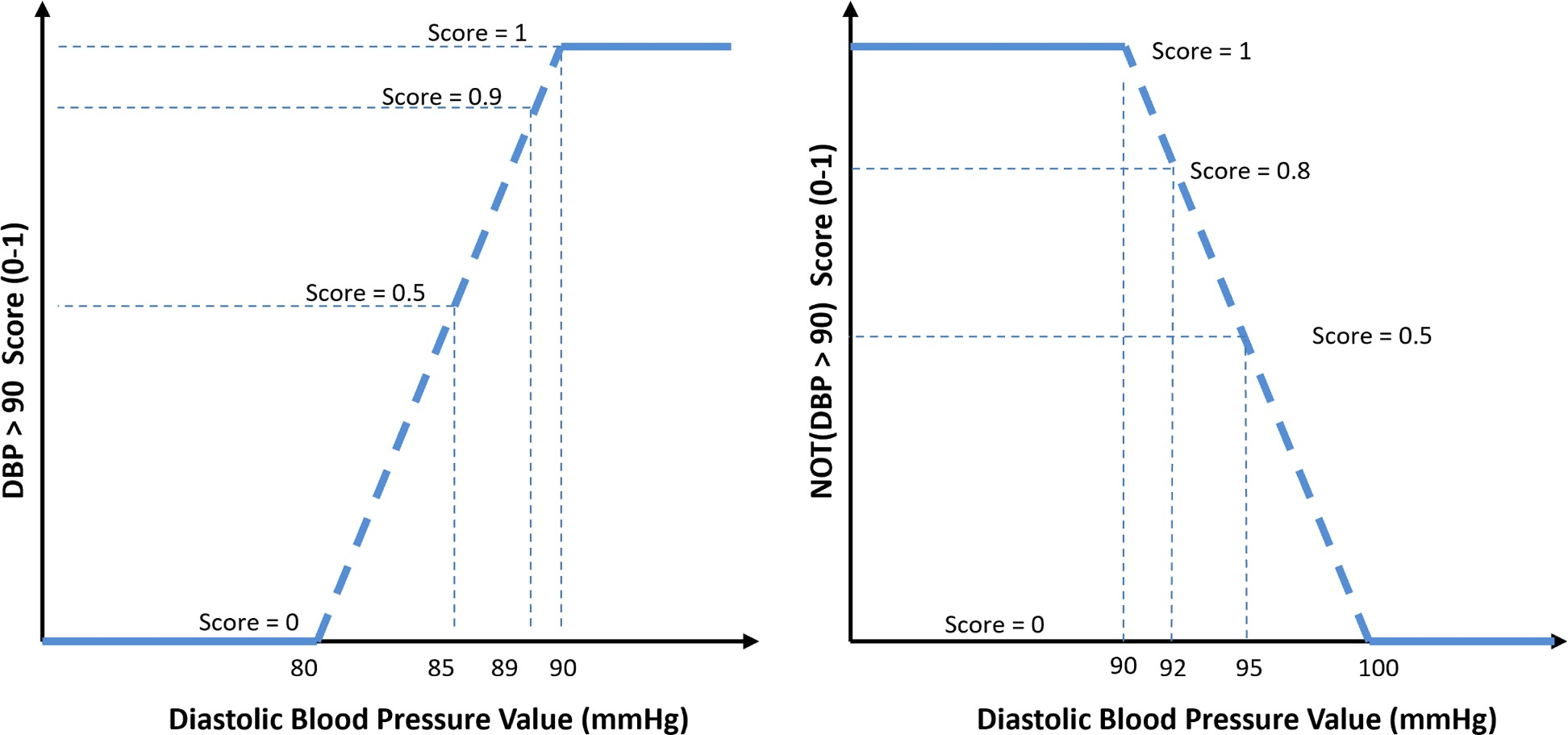

Supplement: S8 Fig — The left graph displays the evaluation of the constraint DBP > 90mmHg, with deviation interval of 10mmHg. The right side displays the evaluation of the false-value of the same constraint, i.e., NOT(DBP > 90mmHg), assuming the same deviation interval. (TIF) [file pone.0303542.s009.tif]
